# Data relating to fate and transport of organophosphate ester flame retardants in indoor air and dust from Nepal

**DOI:** 10.1016/j.dib.2019.104287

**Published:** 2019-08-07

**Authors:** Ishwar Chandra Yadav, Ningombam Linthoingambi Devi

**Affiliations:** a Department of International Environmental and Agricultural Science (IEAS), Tokyo University of Agriculture and Technology (TUAT) 3-5-8, Saiwai-Cho, Fuchu-Shi, Tokyo 1838509, Japan; b Department of Environmental Sciences, Central University of South Bihar, SH-7, Gaya-Panchanpur, Post-Fatehpur, P.S-Tekari, District-Gaya, 824236, Bihar, India

**Keywords:** Flame retardants, Indoor air, House dust, Nepal, Consumer materials

## Abstract

The ubiquitous occurrence of organophosphate ester flame retardants (OPFRs) may pose a threat to human health. Most of the OPFRs are suspected to be carcinogenic, neurotoxic and can cause fertility and reproductive effects (World Health Organization, 2000) and (Van der Veen and De Boer, 2012). Although a number of researches have detailed high level of organophosphate ester flame retardant worldwide, unfortunately Nepal has never been part of any global/regional monitoring campaign. This data article presents the concentration of eight different compounds of organophosphate ester flame retardants (OPFRs) measured in indoor air and house dust. Thirty four indoor air and 28 house dust samples were collected from four major cities (Kathmandu, Pokhara, Birgunj, and Biratnagar) of Nepal to investigate the contamination level and distribution pattern of OPFR. The median concentration and relative contribution of individual OPFR has been also discussed (Yadav et al., 2017).

**Table:** Specifications Table

Subject area	Environmental Science
More specific subject area	Geochemistry and Ecotoxicology
Type of data	Table, graph, figure
How data was acquired	Gas chromatography (Agilent 7890A) coupled with mass spectrometry (Agilent 7000A)
Data format	Raw and analyzed data
Experimental factors	The indoor sample was collected by passive air sampler installed in indoor for 60 days, while dust samples were collected by sweeping of the kitchen room, study room, bed room, living room, office and passage of concerned household
Experimental features	Indoor air and house dust samples were collected from four major cities (Kathmandu, Pokhara, Birgunj, and Biratnagar) of Nepal. Both the air and dust samples were pretreated, extracted and cleaned up following standard protocol. Later, the purified eluent were analyzed for OPFR compounds using Gas Chromatography-Mass Spectrometry.
Data source location	Nepal: Kathmandu (KTM), Pokhara (PKH), Birgunj (BRG), and Biratnagar (BRT)
Data accessibility	Data is given in this article
Related research article	Yadav, I.C., Devi, N.L., Zhong, G., Li, J., Zhang, G., Covaci, A. 2017.Occurrence and fate of Organophosphate Ester Flame Retardants and Plasticizers in indoor air and dust of Nepal: Implication for human exposure. Environmental Pollution 229, 668–678 [3]

**Table:** 

**Value of the Data** • This data provide baseline and preliminary data on contamination level of OPFRs in urban environment of Nepal • This data can be useful for stakeholder/policymaker and health official to formulate the remediation implementation plan • Further, this data can be also used by researcher/scientist/investigator who conducts research on FRs • Due to lack of scientific research and technological advancement, the information about OPFRs is very limited in case of Nepal, hence this data will fill the data-gap

## 1 Data

The concentration of eight different compounds of OPFRs measured in indoor air per passive air sampler (PAS) at individual sampling sites in each Nepalese city is given in Table 3. This initial concentration of OPFRs was converted into air concentration (ng/m^3^) using an uptake rate of 3.3 m^3^/day and is shown in Table 4. Table 5 discusses the level of OPFR compounds measured in house dust samples from four cities of Nepal. Fig. 2 describe the map of Nepal showing sampling location. The relative contribution of each OPFR chemicals has been shown in Fig. 2. Fig. 3 discusses the site-wide compositional pattern of OPFR chemicals in air and dust.

**Table 1:** Full name and GS-MS parameter of OPFRs.

Acronym	Full name	CAS No.	Chemical formula	Mol. Wt.	Quantifier/Qualifier	RT
TNBP	Tri-*n*-butyl phosphate	126-73-8	C_12_H_27_O_4_P	266.3	155/99	7.063
TCEP	Tris (2-chloroethyl)phosphate	115-96-8	C_6_H_12_Cl_3_O_4_P	285.5	249/143	7.696
TCIPP-1	Tris (1-chloro-2-propyl) phosphate (mix of three isomers)	13674-84-5	C_9_H_18_Cl_3_O_4_P	327.6	125/277	7.877
TCIPP-2					125/277	7.952
TCIPP-3					125/277	8.022
TDCIPP	Tris (1,3-dichloropropyl) phosphate	13674-87-8	C_9_H_15_Cl_6_O_4_P	430.9	191/381	12.210
TPHP	Triphenyl phosphate	115-86-6	C_18_H_15_O_4_P	326.3	170/228	13.107
EHDPHP	2-Ethylhexyl diphenyl phosphate	1241-94-7	C_20_H_27_O_4_P	362.4	251/170	13.329
TEHP	Tri (2-ethylhexyl)phosphate	78-42-2	C_24_H_51_O_4_P	434.6	113/211	13.592
TMPP-1	Tri-cresyl phosphate (mix of three isomers)	1330-78-5	C_21_H_21_O_4_P	368.4	243/170	16.020
TMPP-2					243/170	16.400
TMPP-3					243/170	16.790
TCEP-d12	deuterated tris (2-chloroethyl) phosphate	1276500-47-0	C_6_H_12_Cl_3_O_4_P	297.5	261/148	7.635
HMB	Hexamethylbenzene	87-85-4	C_12_H_18_	162.3	162/147	6.330

**Table 2:** Level of average OPFR and RSD detected in blank samples of air, dust, soil and sediments.

OPFRs	Air blank (ng/m^3^)				Dust blank (ng/g)	
	Field (n=3)	RSD (%)	Lab(n=10)	RSD (%)	Lab (n=10)	RSD (%)
TNBP	8.10	4.10	2.38	1.50	3.12	1.11
TCEP	6.33	1.94	3.80	3.41	8.46	3.21
TCIPPs	8.73	2.50	4.62	1.82	11.2	4.30
TDCIPP	9.5	2.61	6.60	2.60	ND	0
TPHP	5.9	1.20	1.72	1.41	0.35	0.01
EHDPHP	5.8	0.07	2.45	1.43	2.68	1.30
TEHP	7.9	4.52	4.9	3.20	ND	0
TMPPs	1.4	0.01	0.82	0.02	16.2	3.91

**Table 3:** Site-wide concentration of OPFR per passive sampler (ng/PAS).

Cities			Sites	TNBP	TCEP	∑TCIPPs	TDCIPP	TPHP	EHDPHP	TEHP	∑TMPP
Kathmandu			KTM-1	40.11	93.93	222.1	14.38	38.50	1761	257.9	545.6
			KTM-2	96.38	174.2	6240	15.30	59.85	857.4	25.21	276.7
			KTM-3	12.17	23.58	20.62	11.99	21.69	3.77	17.51	134.9
			KTM-4	685.2	1019	119.6	15.10	59.05	50.72	26.03	225.3
			KTM-5	35.77	57.01	69.31	11.93	29.51	40.37	3.97	170.5
			KTM-6	39.55	49.25	120.5	16.15	37.52	79.31	346.3	569.9
			KTM-7	28.82	43.18	113.7	12.60	42.41	61.53	126.3	705.1
			KTM-8	21.38	57.67	65.70	14.18	32.06	50.16	124.6	493.7
			KTM-9	80.09	59.34	250.8	16.29	46.46	185.0	196.3	1048
			KTM-10	56.69	49.75	33.88	15.16	35.02	61.91	154.2	287.7
Pokhara			PKH-1	47.82	87.08	74.27	13.65	83.46	28.56	262.4	498.7
			PKH-2	29.47	113.6	58.00	16.66	24.19	153.0	436.9	644.7
			PKH-3	54.55	184.5	2790	17.44	78.33	202.3	446.2	757.6
			PKH-4	26.09	136.1	90.30	13.44	111.2	91.78	311.8	466.0
			PKH-5	45.44	151.3	168.8	13.28	86.51	79.78	363.1	576.5
			PKH-6	30.20	85.49	97.66	11.87	76.65	128.9	85.28	686.9
			PKH-7	45.94	54.53	189.3	13.24	217.5	398.1	418.2	622.2
			PKH-8	610.8	57.78	1112	13.35	63.77	184.2	141.0	447.3
Birgunj			BRG-1	24.05	81.18	102.8	15.91	29.08	41.36	177.0	822.6
			BRG-2	41.70	92.34	87.12	11.19	30.87	26.08	9.66	390.1
			BRG-3	19.35	82.53	296.5	12.34	45.58	80.60	880.3	1740
			BRG-4	35.87	61.63	176.4	11.95	45.39	83.23	241.7	392.4
			BRG-5	33.91	79.32	84.75	12.02	39.88	22.41	106.9	243.4
			BRG-6	28.54	49.94	38.11	11.33	33.82	28.81	11.45	379.9
			BRG-7	18.79	38.29	32.58	13.95	30.39	27.96	192.8	339.2
			BRG-8	8.120	9.10	19.91	2.870	48.93	2.78	7.92	0.810
Biratnagar			BRT-1	104.6	56.24	91.90	12.13	116.3	111.7	227.5	875.4
			BRT-2	53.50	118.1	194.2	11.87	228.3	267.8	383.2	670.7
			BRT-3	62.87	68.96	225.4	12.59	100.5	155.7	4.85	822.4
			BRT-4	42.66	44.35	379.7	12.54	124.4	111.0	81.21	664.8
			BRT-5	116.7	61.83	129.5	11.40	28.17	37.54	61.55	307.9
			BRT-6	82.30	113.1	322.8	16.33	65.94	174.3	158.9	1086
			BRT-7	43.58	67.99	178.3	12.37	45.58	60.16	12.12	384.1
			BRT-8	22.84	31.35	152.1	11.72	30.69	87.02	304.1	491.7

**Table 4:** Concentration of OPFRs (ng/m^3^) in indoor air from four Nepalese cities.

Cities	Sites	TNBP	TCEP	TCIPPs	TDCIPP	TPHP	EHDPHP	TEHP	TMPPs
Kathmandu	KTM-1	0.20	0.47	1.12	0.07	0.19	8.90	1.30	2.76
	KTM-2	0.49	0.88	31.52	0.08	0.30	4.33	0.13	1.40
	KTM-3	0.06	0.12	0.10	0.06	0.11	0.02	0.09	0.68
	KTM-4	3.46	5.15	0.60	0.08	0.30	0.26	0.13	1.14
	KTM-5	0.18	0.29	0.35	0.06	0.15	0.20	0.02	0.86
	KTM-6	0.20	0.25	0.61	0.08	0.19	0.40	1.75	2.88
	KTM-7	0.15	0.22	0.57	0.06	0.21	0.31	0.64	3.56
	KTM-8	0.11	0.29	0.33	0.07	0.16	0.25	0.63	2.49
	KTM-9	0.40	0.30	1.27	0.08	0.23	0.93	0.99	5.29
	KTM-10	0.29	0.25	0.17	0.08	0.18	0.31	0.78	1.45
Pokhara	PKH-1	0.24	0.44	0.38	0.07	0.42	0.14	1.33	2.52
	PKH-2	0.15	0.57	0.29	0.08	0.12	0.77	2.21	3.26
	PKH-3	0.28	0.93	14.09	0.09	0.40	1.02	2.25	3.83
	PKH-4	0.13	0.69	0.46	0.07	0.56	0.46	1.57	2.35
	PKH-5	0.23	0.76	0.85	0.07	0.44	0.40	1.83	2.91
	PKH-6	0.15	0.43	0.49	0.06	0.39	0.65	0.43	3.47
	PKH-7	0.23	0.28	0.96	0.07	1.10	2.01	2.11	3.14
	PKH-8	3.09	0.29	5.62	0.07	0.32	0.93	0.71	2.26
Birgunj	BRG-1	0.12	0.41	0.52	0.08	0.15	0.21	0.89	4.15
	BRG-2	0.21	0.47	0.44	0.06	0.16	0.13	0.05	1.97
	BRG-3	0.10	0.42	1.50	0.06	0.23	0.41	4.45	8.79
	BRG-4	0.18	0.31	0.89	0.06	0.23	0.42	1.22	1.98
	BRG-5	0.17	0.40	0.43	0.06	0.20	0.11	0.54	1.23
	BRG-6	0.14	0.25	0.19	0.06	0.17	0.15	0.06	1.92
	BRG-7	0.09	0.19	0.16	0.07	0.15	0.14	0.97	1.71
	BRG-8	0.04	0.05	0.10	0.01	0.25	0.01	0.04	0.00
Biratnagar	BRT-1	0.53	0.28	0.46	0.06	0.59	0.56	1.15	4.42
	BRT-2	0.27	0.60	0.98	0.06	1.15	1.35	1.94	3.39
	BRT-3	0.32	0.35	1.14	0.06	0.51	0.79	0.02	4.15
	BRT-4	0.22	0.22	1.92	0.06	0.63	0.56	0.41	3.36
	BRT-5	0.59	0.31	0.65	0.06	0.14	0.19	0.31	1.56
	BRT-6	0.42	0.57	1.63	0.08	0.33	0.88	0.80	5.49
	BRT-7	0.22	0.34	0.90	0.06	0.23	0.30	0.06	1.94
	BRT-8	0.12	0.16	0.77	0.06	0.16	0.44	1.54	2.48

**Table 5:** Site-wide concentration of OPFR in house dust (ng/g) from four major cities of Nepal.

Cities	Sites	TNBP	TCEP	TCIPPs	TDCIPP	TPHP	EHDPHP	TEHP	TMPPs
Kathmandu	KTD-1	25.0	13.0	100.9	16.8	477.4	156.2	41.0	3069.8
	KTD-2	22.7	29.7	136.6	1417.0	159.7	116.7	163.3	530.9
	KTD-4	17.4	22.3	35.3	20.5	69.8	90.0	28.5	1128.0
	KTD-4	19.3	22.4	45.0	26.6	72.7	87.4	32.5	2050.0
	KTD-5	27.3	20.6	59.8	28.4	92.8	60.4	75.1	341.8
	KTD-6	25.1	69.1	63.9	26.6	12.2	85.3	42.0	1206.2
	KTD-7	18.7	24.1	38.1	22.1	75.4	97.2	30.8	1218.2
Pokhara	PKD-1	45.1	11.2	125.0	22.0	190.6	530.5	749.5	220.3
	PKD-2	72.2	19.4	48.1	10.2	1521.3	219.1	238.3	1009.2
	PKD-3	68.9	37.5	96.3	50.0	251.9	257.7	96.4	510.3
	PKD-4	116.4	8.2	804.9	26.4	159.5	214.6	167.4	794.8
	PKD-5	45.9	5.9	71.4	8.2	64.7	75.7	45.8	294.4
	PKD-6	45.0	6.0	42.3	9.2	46.7	77.7	31.8	206.7
	PKD-7	47.0	1.3	195.0	55.2	26.5	50.6	31.8	235.2
Birgunj	BRD-1	47.3	4.5	59.0	11.3	323.3	287.7	182.4	1218.0
	BRD-2	18.3	1.5	57.8	1.1	17.8	26.3	26.3	70.1
	BRD-3	19.9	13.3	103.3	8.2	9.8	27.9	46.1	110.0
	BRD-4	19.4	3.5	51.1	11.0	23.2	39.7	33.1	151.6
	BRD-5	19.3	20.8	119.3	28.2	138.1	56.6	32.6	478.8
	BRD-6	18.8	27.1	166.4	22.6	141.4	52.5	30.6	449.4
	BRD-7	22.6	32.5	199.7	27.2	169.7	63.0	36.7	539.3
Biratnagar	BID-1	21.5	9.2	36.1	3.2	60.9	107.8	159.9	362.0
	BID-2	18.5	37.4	48.8	24.6	50.6	49.0	117.9	857.2
	BID-3	30.7	5.2	29.6	8.3	57.5	49.9	41.7	201.5
	BID-4	14.8	18.1	33.7	30.8	67.3	39.1	33.2	1227.0
	BID-5	20.0	0.1	34.9	1.0	11.7	32.0	26.4	105.6
	BID-6	21.8	3.7	34.8	5.7	3669.3	85.9	44.3	323.5
	BID-7	19.6	39.7	51.7	26.1	53.7	52.0	125.0	908.7

**Fig. 1:**
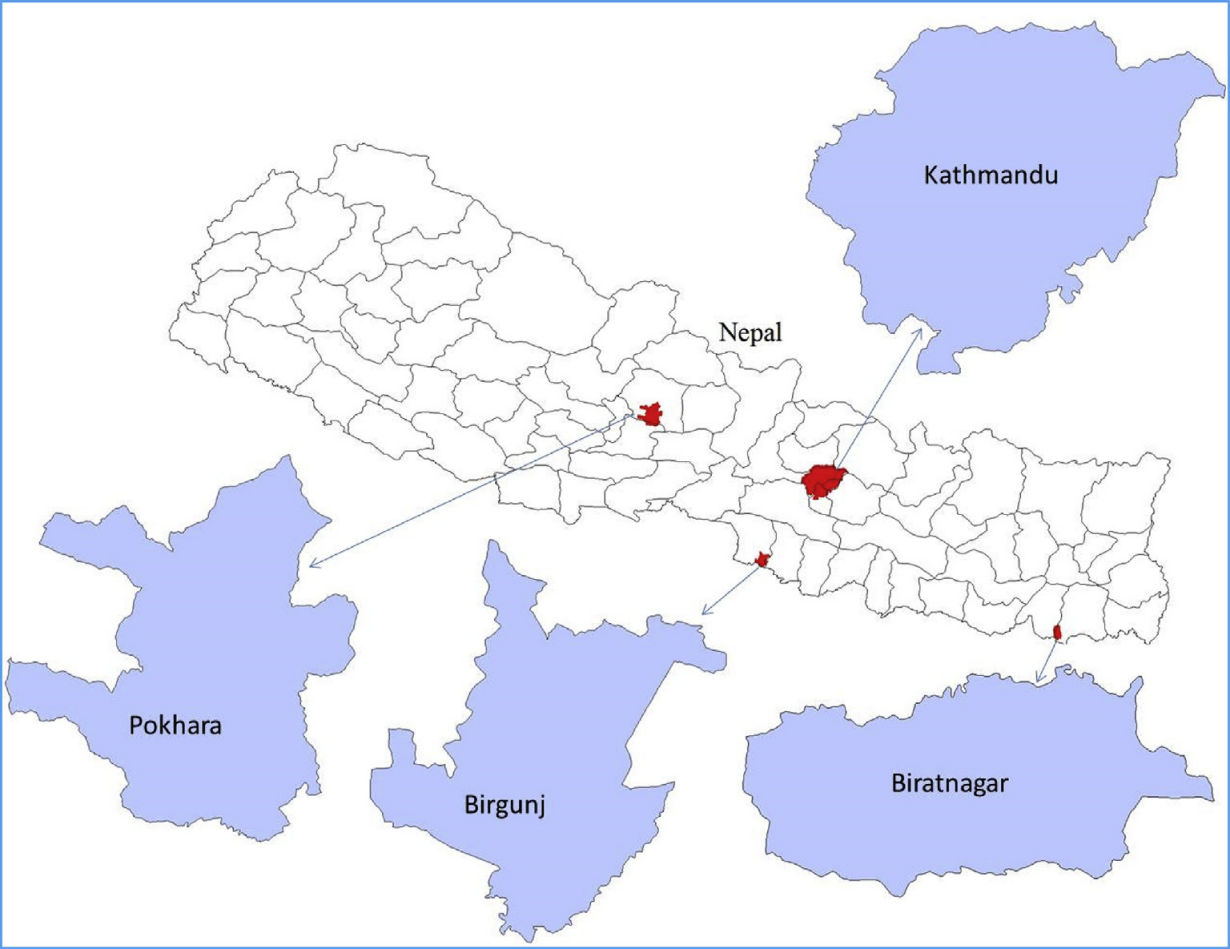
Map of Nepal showing sampling area location.

**Fig. 2:**
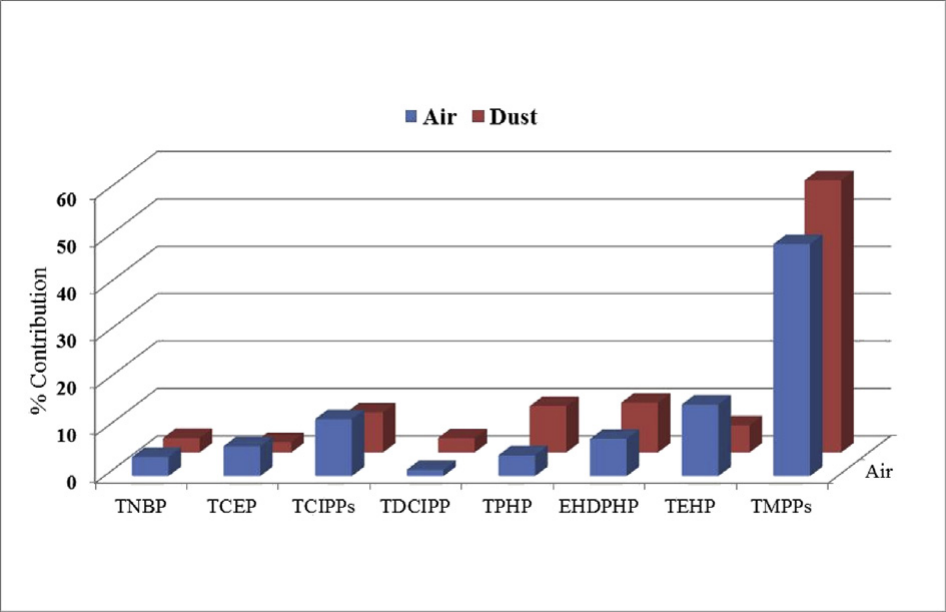
Relative contributions of OPFRs in air and dust matrix from four cities of Nepal.

**Fig. 3:**
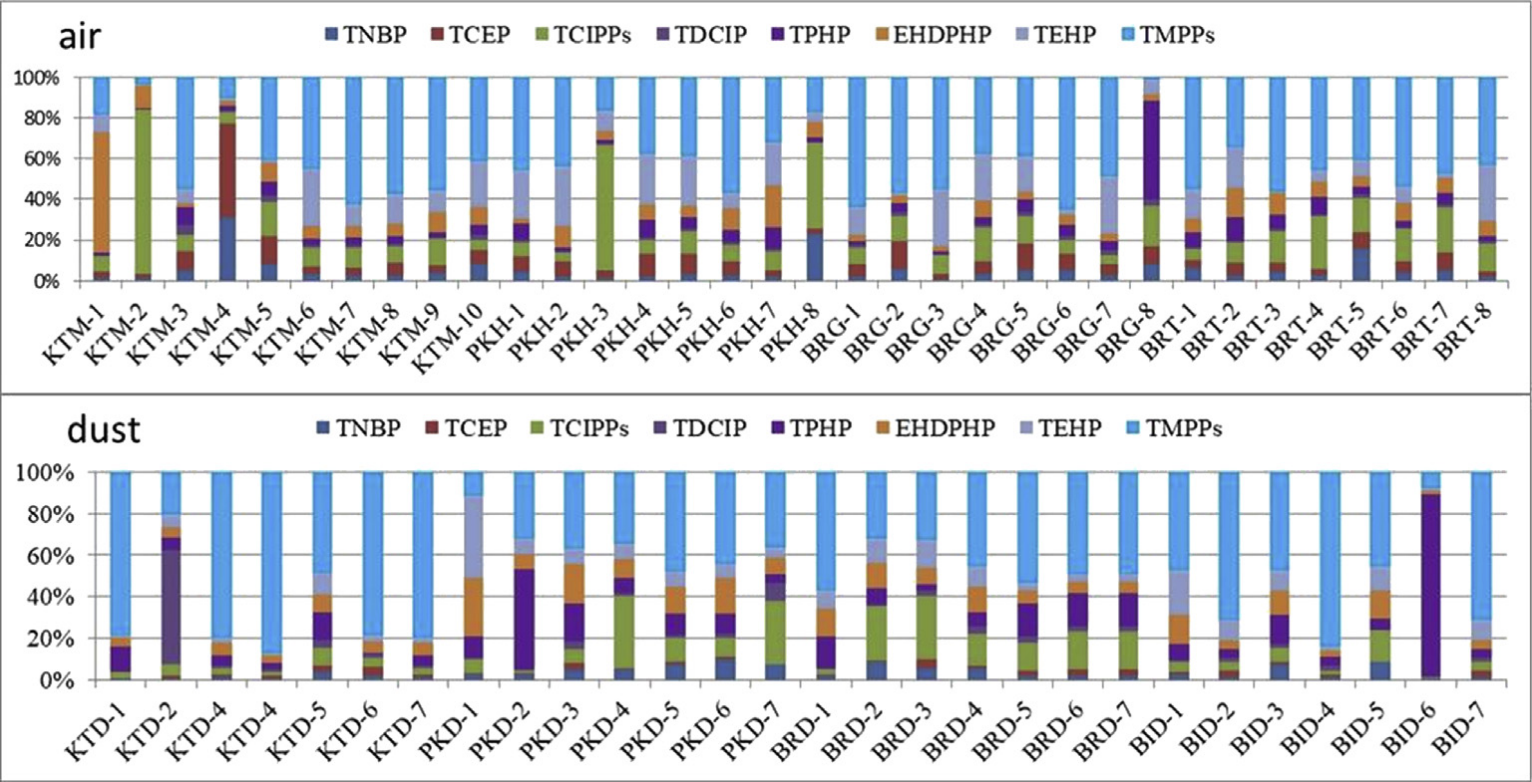
Site-specific composition profiles of OPFRs in air and dust (top to bottom) samples from four Nepalese cities.

## 2 Experimental design, materials, and methods

### 2.1 Sampling area

Four major cities of Nepal including one metropolitan city (Kathmandu) and 3 sub metropolitan cities (Pokhara, Birgunj and Biratnagar) were selected for the collection of air, dust, soil and sediment samples. Different categories of samples were collected from different environmental matrix during August–October 2014. A location map of sampling area has been given in Fig. 1.

### 2.2 Air sampling

A total of 34 polyurethane foams mounted in passive air samplers (PUF-PAS) (8 PAS each at Pokhara, Birgunj, Biratnagar and 10 PAS at Kathmandu) were deployed in 34 different household of selected cities. Prior to deployment, each PUF disk (Diameter 14.0 cm; Thickness 1.30 cm; Surface area, 365 cm^2^; Volume, 200 cm^3^; Density, 0.0170 g/cm^3^) was pre-cleaned by Soxhlet extraction with acetone and dichloromethane (DCM) each for 48 h. After the exposure, all PUF samples were transported to the laboratory immediately and stored at −20 °C until analysis. The details about PAS procedure for collection of air samples has been described elsewhere [2].

### 2.3 Dust sampling

Twenty eight dust (7 each) samples were collected from indoor environment representing residential, commercial, office premises, public places, intensive traffic zone, airport, industrial area and occupational areas. Samples were collected by sweeping of kitchen room, study room, bed room, living room, office and passage of concerned household. About 50 g of dust samples were collected and packed in zipper bag before transporting to the laboratory. Dust samples were sieved with mess size of 500μm and stored at −20 °C until analysis.

### 2.4 Sample preparation

Extraction and cleanup: PUF disks or/and freeze-dried dusts, soils and sediments samples were spiked with 1000 ng of deuterated tris (2-chloroethyl) phosphate (TCEP-d12) as surrogate standard and were Soxhlet extracted with DCM for 24 h. Copper granules were added to the round bottle flask before extraction to remove the elemental sulphur present in dust. The sample extract was concentrated by rotary evaporator (Heildolph 4000, Germany) and were solvent exchanged to hexane with a volume of 0.5 ml. The extract was passed through Supelclean Envi Florisil SPE column tubes 6 ml (1g) (SUPELCO, USA). Prior to fractionation, Florisil® cartridges were prewashed with 6 ml ethyl acetate, 6 ml hexane/DCM (8:2, v/v), and 10 ml hexane to clean and condition the adsorbent. After the extract was transferred to the SPE column, first fraction was eluted with 6 ml 8:2 Hex: DCM and was discarded. The second fraction that contained target OPFRs were eluted with 20ml ethyl acetate, evaporated until dryness under constant nitrogen flow and the residue was re-dissolved in 200 μL of *iso*-octane. The resulting fraction was transferred to vials for GC-MS analysis. Prior to GCMS injection, a known amount (1000 ng) of hexamethyl benzene (HMB) were added as internal standard for quantification.

### 2.5 GC-MS analysis

Eight target OPFRs (TCEP, TCIPPs: mix of three isomers, TDCIPP, TNBP, TEHP, TPHP, EHDPHP and TMPPs: mix of three isomers) were analyzed using GC-EI-MS (Agilent GC 7890A coupled with 7000A Triple quadrupole coupled MSD), with a DB5-MS capillary column (30 m × 0.25 mm i.d. × 0.25 μm film thickness). One μL of sample was injected in split less mode and temperature of injector was 295 °C. Helium was used as carrier gas at the flow rate of 1 mL min^−1^. The temperature of transfer line and ion source was maintained at 280 °C and 230 °C, respectively. The GC oven temperature started at 60 °C for 1 min, increased to 220 °C at a rate of 30 °C min^−1^ (held for 0 min), then to 300 °C at a rate of 5 °C min^−1^ (held for 15 min). The specific parameters for the target compounds were shown in Table 1.

### 2.6 QA/QC

Since OPFRs are ubiquitous to indoor environment [1], all glassware used for these experiments was solvent rinsed and baked at 450 °C before use. Three field blank (only for air sample) and ten laboratory blank each for air and dust were extracted and analyzed together with samples to assess the possible contamination of the samples. The level of OPFRs detected in laboratory blank ranged from 1.4 to 9.5ng/m^3^ for air and 0.35–11.2 ng/g for dust, samples (Table 2). The method detection limits (MDLs) is the mean plus 3 times standard deviation of all the blanks samples. When the compounds were detected in blank, the MDL was calculated as 3 times signal to noise ratio obtained from lowest spiked standard. The MDLs of OPFRs ranged from 0.88 to 14.4 ng/m^3^ and 0.38–27.93 ng/g, for air and dust samples, respectively. The average recovery of surrogate standard (TCEP-d12) was 80–101% and 108–124% for air and dust, respectively. The concentrations of target OPFRs were blank corrected, but not corrected for recovery.

### 2.7 Conversion of initial concentration of OPFR sequester in PUF/PAS to air concentration (ng/m^3^)

The initial atmospheric concentration of OPFRs were converted in air concentration (ng/m^3^) by dividing the sequestered amounts with the product of deployment period and the sampling rate of PAS using following equation.

$C_{\text{air}}=C_{\text{PUF}}/\text{Rt}$

Where, C_air_ is the concentration of OPFR in air over t (days) deployment period. C_PUF_ is the concentration of OPFR sequestered in PUF disk. R is the sampling uptake rate. We used an average uptake rate of 3.3m^3^/d for calculating the concentrations of OPFR in air sample as suggested by Liu et al. [4]. Fig. 2, Fig. 3 represent the relative contribution and site specific abundance of individual OPFR compound. All the PAS were simultaneously deployed for 60 days from August–October 2014.
